# Identification of the Feline Humoral Immune Response to *Bartonella henselae* Infection by Protein Microarray

**DOI:** 10.1371/journal.pone.0011447

**Published:** 2010-07-06

**Authors:** Adam Vigil, Rocio Ortega, Aarti Jain, Rie Nakajima-Sasaki, Xiaolin Tan, Bruno B. Chomel, Rickie W. Kasten, Jane E. Koehler, Philip L. Felgner

**Affiliations:** 1 Division of Infectious Diseases, Department of Medicine, University of California Irvine, Irvine, California, United States of America; 2 Department of Population Health and Reproduction, School of Veterinary Medicine, University of California Davis, Davis, California, United States of America; 3 Division of Infectious Diseases, Department of Medicine, University of California San Francisco, San Francisco, California, United States of America; Federal University of São Paulo, Brazil

## Abstract

**Background:**

*Bartonella henselae* is the zoonotic agent of cat scratch disease and causes potentially fatal infections in immunocompromised patients. Understanding the complex interactions between the host's immune system and bacterial pathogens is central to the field of infectious diseases and to the development of effective diagnostics and vaccines.

**Methodology:**

We report the development of a microarray comprised of proteins expressed from 96% (1433/1493) of the predicted ORFs encoded by the genome of the zoonotic pathogen *Bartonella henselae*. The array was probed with a collection of 62 uninfected, 62 infected, and 8 “specific-pathogen free” naïve cat sera, to profile the antibody repertoire elicited during natural *Bartonella henselae* infection.

**Conclusions:**

We found that 7.3% of the *B. henselae* proteins on the microarray were seroreactive and that seroreactivity was not evenly distributed between predicted protein function or subcellular localization. Membrane proteins were significantly most likely to be seroreactive, although only 23% of the membrane proteins were reactive. Conversely, we found that proteins involved in amino acid transport and metabolism were significantly underrepresented and did not contain any seroreactive antigens. Of all seroreactive antigens, 52 were differentially reactive with sera from infected cats, and 53 were equally reactive with sera from infected and uninfected cats. Thirteen of the seroreactive antigens were found to be differentially seroreactive between *B. henselae* type I and type II. Based on these results, we developed a classifier algorithm that was capable of accurately discerning 93% of the infected animals using the microarray platform. The seroreactivity and diagnostic potential of these antigens was then validated on an immunostrip platform, which correctly identified 98% of the infected cats. Our protein microarray platform provides a high-throughput, comprehensive analysis of the feline humoral immune response to natural infection with the alpha-proteobacterium *B. henselae* at an antigen-specific, sera-specific, and genome-wide level. Furthermore, these results provide novel insight and utility in diagnostics, vaccine development, and understanding of host-pathogen interaction.

## Introduction

Controlling *Bartonella* infection in its cat reservoir is integral to preventing cat scratch disease (CSD) in humans. *B. henselae* infection is mainly asymptomatic in cats, but has been associated with kidney disease and urinary tract infections, stomatitis, and lymphadenopathy [1]. The prevalence of *Bartonella* infection in cats ranges from 25% to as high as 41% throughout the world [2]. Infected cats can have bacterial titers of >10^6^ colony forming units (CFU)/ml of blood and can remain bacteremic for several months to several years. Cats that are bacteremic, especially with high titers, are more likely to infect humans by scratches or bites. Although antibiotic treatment of infected cats has been associated with reduction of bacteremia levels, treatment does not appear to be sufficient to completely eradicate *B. henselae* from the blood stream [3]. Indeed, treatment can result in increased transmission of *B. henselae* to humans during attempts to administer antibiotics pills to uncooperative, infected cats.

Preventing initial infection of cats by vaccination is a potential strategy for limiting *B. henselae* infections in humans. With an estimated 90 million pet cats in the US and a predicted 8–20 million cats with chronic bacteremia, prevention and reduction of morbidity in humans from CSD could be achieved through extensive cat vaccination programs [4]. Profiling the feline host antibody response to *B. henselae* infection is central to diagnostics development and the identification of potential subunit vaccine candidates. Importantly, there are two major genotypes of *B. henselae* that can cause CSD in humans: types I (Houston); and II (Marseille) [5]. Cats are most often infected with one or the other type, but some cats are co-infected with both types, and both types can be transmitted to humans from pets [5]. Thus, establishing and comparing the host immune profile to infection with both types may be necessary for optimizing candidate antigen selection to prevent feline infection with type I and type II.

We previously developed a protein microarray technology that allows construction of the complete predicted proteome of a microorganism [6], [7], [8], [9], [10]. Utilization of arrays constructed from *in vitro* transcription reactions can identify the repertoire of seroreactive antibodies to proteins encoded by an infectious agent. These arrays are limited to detection of antibodies against recombinant proteins and would not detect post-translational modifications and non-protein antigens [11]. However, these arrays can be utilized to address basic questions about the pattern of the host humoral immune response to infectious agents [12], [13], [14], and to identify individual antigens that could be used as diagnostic reagents or for inclusion in vaccines [6], [15]. The data derived from these studies can also be used to evaluate and improve the accuracy of *in silico* predictions of seroreactive antigens, and can provide a more detailed understanding of the adaptive immune response to infection. In this study, we developed a *B. henselae* genome-wide protein array and used the arrays to profile the antibody response in naturally infected cats and uninfected cats.

## Materials and Methods

### Bacterial strains

DNA extracted from *B. henselae* wild type strain JK33R was used for template DNA from which all ORFs were amplified prior to cloning. This *B. henselae* strain was isolated from the blood of an AIDS patient with bacillary angiomatosis and was cryopreserved after only several passages on agar. JK33R retains the rough colony phenotype characteristic of primary *B. henselae* isolates obtained from human and feline blood.

### Cat serum samples

All procedures involving animals followed NIH protocols and were approved by and performed according to guidelines of the Institutional Animal Care and Use Committee of University of California, Davis. Serum samples were collected in 2008 from 124 cats housed in two shelters, one in California (Sacramento) and one in Michigan (Muskegon). These sera were tested for the presence of *Bartonella* antibodies at two serial dilutions 1∶32 and 1∶64 by two of the authors (BBC, RWK). A cat was reported serologically positive when the scoring on a scale from 0 to 4 was ≥2 at the 1∶64 dilution. Uninfected cats had an IFA score range of 0.3±0.5 for *B. henselae* and 0.5±0.6 for *B. clarridgeiae* at the 1∶64 dilution and the IFA score range at the 1∶64 dilution for infected cats was 2.0±1.3 for *B. henselae* and 2.8±0.8 for *B. clarridgeiae*. Of these cats, 62 were identified as *Bartonella* blood culture negative and seronegative for *Bartonella* IgG antibodies by an IFA test [16]. Another 62 cats were determined to be either seropositive (IFA titer ≥1∶64) or both IFA and culture positive (24 of the 62 seropositive cats were also culture positive). Similarly, serum samples from 8 *Bartonella*-uninfected (“specific pathogen free [SPF]”) cats were submitted for testing and were both culture and IFA negative. Cats ranged in age from ∼3 months to adult (average uninfected cat age was approximately 19.9 months old with a standard deviation of 19.7 months; average infected cat age was approximately 19.4 months old with a standard deviation of 20.3).

### PCR amplification of linear acceptor vector

Each predicted open reading frame (ORF) from the *B. henselae* genome sequence was amplified from *B. henselae* JK33 strain DNA, and was cloned into the pXT7 vector using a high-throughput PCR cloning method previously described [6]. The pXT7 plasmid (3.2 kb, KanR) encodes an N-terminal 10 x histidine (HIS) tag and a C-terminal hemagglutinin (HA) tag. pXT7 (10 µg) was linearized with *Bam*HI (0.1 µg/µl DNA, 0.1 mg/mL BSA, 0.2 U/µl *Bam*HI, Invitrogen) overnight at 37°C. The digest was purified using a PCR purification kit (Qiagen, Valencia, CA), quantified using a NanoDrop (Thermo Scientific), and verified by agarose gel electrophoresis. PCR was used to generate the linear acceptor vector in 50 µl PCR reactions with 0.5 µM of each primer (CTACCCATACGATGTTCCGGATTAC and CTCGAGCATATGCTTGTCGTCGTCG). PCR was performed using 0.02 U/µl AccuPrime Taq DNA polymerase (Invitrogen), 0.2 mM of each dNTP, and 1 ng pXT7 diluted in AccuPrime Buffer II. The following PCR conditions were used: 95°C for 5 min; 30 cycles of 95°C for 0.5 min, 50°C for 0.5 min, 72°C for 3.5 min; and a final extension at 72°C for 10 min.

### Open reading frame cloning

Primers were designed to all 1,493 ORFs that did not contain an internal stop codon (1493/1612), as predicted from the genome sequence of the *B. henselae* Houston-1 strain (BX897699.1) [17]. PCR primers were designed for the 5′ and 3′ ends of each ORF, with the addition of a 20 bp homologous recombination “adapter” sequence (ACGACAAGCATATGCTCGAG and TCCGGAACATCGTATGGGTA respectively). The adapter sequences, which become incorporated into the termini flanking the amplified gene, are homologous to the cloning sites of the linearized T7 expression vector pXT7. ORFs that are larger than 3000 bp were split into smaller fragments with 150 bp overlap for efficient amplification. PCR reactions were prepared using 0.02 U/µl AccuPrime Taq DNA polymerase (Invitrogen), 0.2 mM of each dNTP, diluted in Buffer II, with 2.5 ng of *B. henselae* template DNA, using the following conditions: 95°C for 2 min; 30 cycles of 95°C for 0.33 min, 55°C for 0.25 min, 50°C for 0.25 min, 68°C for 3 min; and a final extension of 68°C for 10 min. All *B. henselae* ORF-PCR reactions were confirmed by gel electrophoresis for correct insert size prior to cloning into pXT7.

### High-throughput recombination cloning

All ORFs amplified from *B. henselae* strain JK33R DNA were cloned into the plasmid expression vector pXT7 using a high-throughput PCR recombination cloning method previously developed in our laboratory [6]. Linearized pXT7 was diluted to 10 ng/µl, mixed with 1 µl of *B. henselae* ORF PCR reaction mixture at a volume ratio of 4∶1, and incubated on ice for 2 min, followed by addition of 10 µl of competent *E. coli* DH5α cells (MCLabs). Reactions were mixed, incubated on ice for 30 min, heat shocked at 42°C for 1 min, and chilled on ice for 2 min. 250 µl of SOC media was added and cells were incubated for 1 hr at 37°C. The entire reaction mixture was added to 1.5 ml of LB medium supplemented with 50 µg/mL of kanamycin, and incubated overnight at 37°C with shaking. Plasmids were isolated using QIAprep 96 Turbo kits (Qiagen, Valencia, CA) without colony selection. Minipreps of all attempted clones were analyzed by agarose gel electrophoresis to confirm insert size. 25% of all clones were confirmed for insert size by PCR using ORF sequence-specific primers. An additional 25% of all clones were selected at random and sequenced in both directions. Sequences were analyzed for fidelity, orientation, and for mutation in the overlapping region of the homologous recombination sites. The cloning efficiency for all amplified ORFs was 98.4%, resulting in 1520 plasmids encoding proteins from 1464 ORFs.

### Protein microarray chip printing

The expression of cloned ORFs was carried out for five hours in *in vitro* transcription-translation (IVTT) reactions (RTS 100 kits, Roche) according to the manufacturer's instructions. Protein microarrays were printed onto nitrocellulose-coated glass FAST slides (Whatman) using an Omni Grid 100 microarray printer (Genomic Solutions). 3.3 µl of 0.2% Tween-20 was mixed with 10 µl of IVTT and transferred to 384-well plates. Plates were centrifuged at 1600× g to pellet any precipitate and remove air bubbles prior to printing. Supernatants were printed immediately without purification, and all ORFs were spotted in duplicate. Data values reported herein represent an average of the pair, unless otherwise mentioned. In addition, each chip was printed with control spots consisting of IVTT reactions without plasmid, purified IgGs, and purified EBNA1 proteins. Protein expression was confirmed using monoclonal anti-polyhistidine (clone His-1, Sigma) and anti-hemagglutinin (clone 3F10, Roche).

### Microarray probing

Feline sera were preabsorbed with *E. coli* lysate prior to array staining, to remove background reactivity to *E. coli* proteins in the IVTT reactions. The sera were diluted to 1∶200 in Protein Array Blocking Buffer (Whatman) containing 15 mg/ml reconstituted *E. coli* lysate (McLabs) and incubated at room temperature for 30 minutes with constant mixing. The arrays were rehydrated in blocking buffer for 30 min and probed with the preabsorbed sera overnight at 4°C with constant agitation. The slides were then washed five times in 10 mM Tris (hydroxymethyl) aminomethane buffer (pH 8.0) containing 0.05% (v/v) Tween-20 (TTBS), and incubated in biotin-conjugated, goat anti-cat immunoglobulin (anti-IgGfcγ, Jackson Immuno Research) diluted 1/200 in blocking buffer. After washing the slides three times in TTBS, bound antibodies were detected by incubation with streptavidin-conjugated SureLight® P-3 (Columbia Biosciences). The slides were then washed three times in TTBS and three times in Tris buffer without Tween-20 followed by a final water wash. The slides were air dried after brief centrifugation and analyzed using a Perkin Elmer ScanArray Express HT microarray scanner.

### Immunostrip assay

Fifteen sequence-confirmed plasmids were expressed in five-hour IVTT reactions, according to the manufacturer's instructions. Proteins were printed on Optitran BA-S 85 0.45 µm Nitrocellulose membrane (Whatman) using a BioJet dispenser (BioDot) at 1 µl/cm, and cut into 3 mm strips. Individual strips were then blocked for 30 minutes in 10% non fat dry milk dissolved in TTBS. Prior to immunostrip probing, cat sera were diluted 1∶250 in 10% nonfat dry milk solution containing 15 mg/ml *E. coli* lysate, and incubated for 30 min with constant mixing at room temperature. Preabsorbed sera were then applied to each strip and incubated overnight at 4°C with gentle mixing. Strips were washed five times in TTBS, and then incubated for 11hour at room temperature in alkaline phosphatase conjugated goat anti-cat immunoglobulin (anti-IgG, Fcγ fragment-specific, Jackson ImmunoResearch), that was diluted to 1∶5000 in TTBS. The strips were then washed three times in TTBS, followed by another three washes in Tris buffer without Tween-20. Reactive bands were visualized by incubating with 1-step Nitro-Blue Tetrazolium Chloride/5-Bromo-4-Chloro-3′-Indolyphosphate p-Toluidine Salt (NBT/BCIP) developing buffer (Thermo Fisher Scientific) for 2.5 minutes at room temperature. The enzymatic reaction was stopped by washing the strips with tap water. Strips were air dried and scanned at 2,400 dpi (Hewlett-Packard scanner). Images were converted to gray scale format by Photoshop and unaltered images are shown. Band intensities were quantified using ImageJ software [18] (found at http://rsbweb.nih.gov/ij/).

### Data and statistical analysis

The protein microarrays used here do not meet the criteria for required deposition under MIAME guidelines [19], and alternatives to standardize protein microarray results are in development to insure that all information can be easily interpreted (description of minimum information about a proteomics experiment [MIAPE] can be found here [20]). Intensities were quantified using QuantArray software utilizing automatic background subtraction for each spot. Proteins were considered to be expressed if either tag's signal intensity was greater than the average signal intensity of the IVTT reaction without plasmid, plus 2.5-times the standard deviation. “No DNA” controls consisting of IVTT reactions without addition of plasmid were averaged and used to subtract background reactivity from the unmanipulated raw data. All results presented are expressed as signal intensity. As previously reported [21], the “vsn” package in the Bioconductor suite (http://Bioconductor.org/) in the R statistical environment (http://www.R-project.org) was used to calculate seroreactivity. In addition to the variance correction, this method calculates maximum likelihood shifting and scaling calibration parameters for different arrays, using known non-differentially expressed spots. This calibration has been shown to minimize experimental effects [22]. We used raw values for the positive and negative controls to calibrate, and then normalize, the entire data set using the vsn package. Differential analysis of the normalized signals was then performed using a Bayes-regularized t-test adapted from Cyber-T for protein arrays [23], [24], [25], [26]. Benjamini-Hochberg p-value adjustments were applied to account for multiple test conditions [27]. All p-values shown are Benjamini-Hochberg corrected for false discovery, unless otherwise noted. Multiple antigen classifiers were built using Support Vector Machines (SVMs). The “e1071” and “ROCR” packages in R were utilized to train the SVMs and to produce receiver operating characteristic curves, respectively.

Computational prediction of transmembrane domains utilized the TMHMM v2.0 software [28] (http://www.cbs.dtu.dk/services/TMHMM/); signal peptide prediction used SignalP v3.0 software [29] (http://www.cbs.dtu.dk/services/SignalP/); cellular location prediction utilized PSORTb v2.0.4 software [30] (http://www.psort.org/psortb/); predicted isolectric point was determined using Swiss Institute of Bioinformatics pI/MW software (http://ca.expasy.org/tools/pi_tool.html); and codon adaptation index (CAI) of each protein was retrieved from JCAT program (http://www.jcat.de)[31]. The COG information utilized can be found at http://www.ncbi.nlm.nih.gov/sutils/coxik.cgi?gi=409. Enrichment statistical analysis was performed in the R environment, using Fisher's exact test. Segmented ORFs were considered seroreactive if any segment was identified as seroreactive.

## Results

### Construction and probing of a *Bartonella henselae* protein microarray with sera from infected and uninfected cats

**Figure 1:**
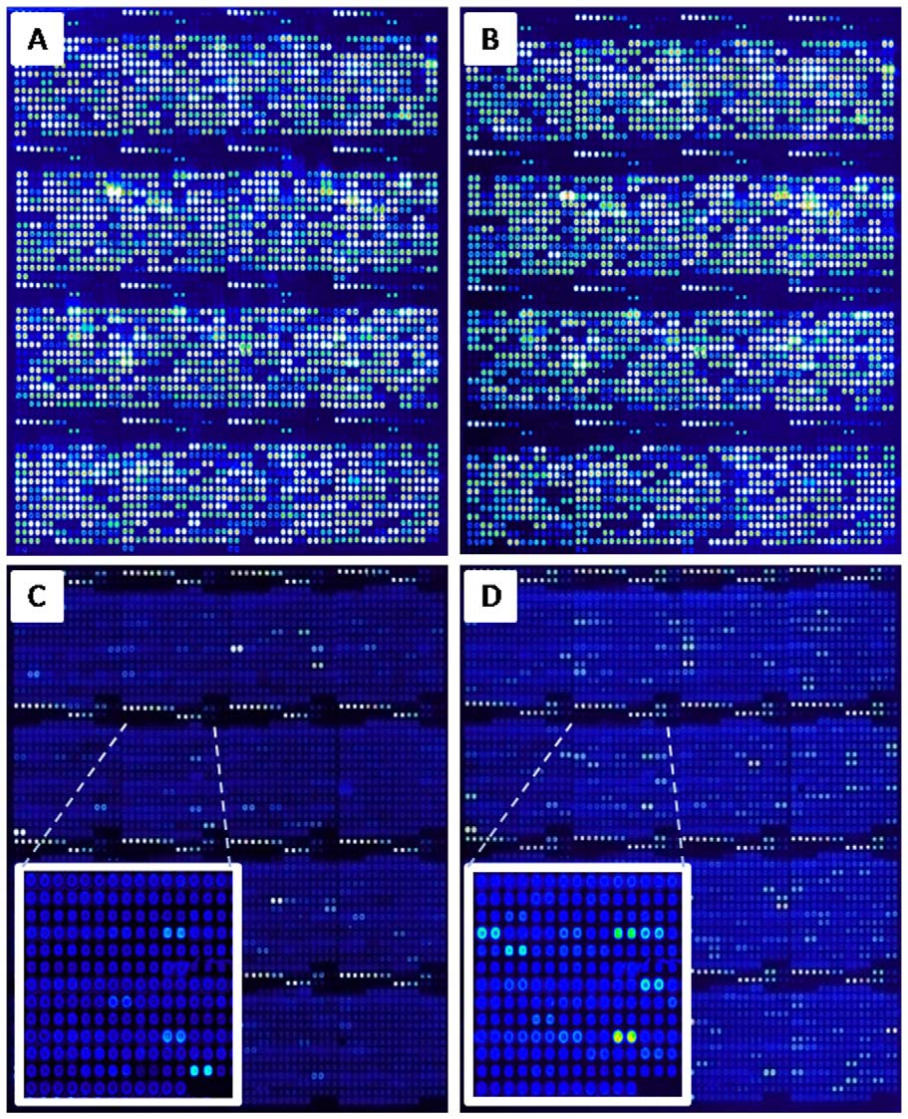
Construction of a *B. henselae* protein microarray. Arrays were printed containing 4032 spots from *B. henselae* proteins, as well as positive and negative control spots. Proteins were printed in duplicates and average signal intensities were calculated. Each array contains positive control spots printed from 6 serial dilutions of purified IgG, 6 serial dilutions of EBNA1 protein, and 6 “No DNA” negative control spots. The array was probed with anti-His antibody (A) or anti-HA antibody (B) as described in Materials and Methods, to confirm the expression and printing of 1433 *B. henselae* ORFs. (C and D) Comparison of arrays probed with naïve serum and positive serum. The arrays were read in a laser confocal scanner, analyzed, and the data normalized as described in Materials and Methods. The signal intensity for each antigen is represented by a rainbow palette of blue, green, red and white, corresponding to increasing signal.

A protein microarray comprised of 4032 spots from 1433 ORFs spotted in duplicate, with positive and negative controls was fabricated, as described in Methods. IVTT expression efficiency was determined by probing against the amino-terminal HIS and carboxy-terminal HA tags for each spot (Fig. 1a and 1b). 95.1% of the *B. henselae* proteins spotted onto the microarray had signals greater than the average of “No-DNA” control reactions plus 2.5 times the standard deviation, and were considered positively expressed. Sera from cats housed in two animal shelters were used to probe the *B. henselae* protein microarray in order to map the feline host anti-*B. henselae* antibody profile after naturally acquired infection. The *B. henselae* protein microarray was probed with a collection of 132 cat sera. Sixty-two cats identified as seropositive or seropositive and culture-positive were compared to 62 cats identified as seronegative and culture negative. The seronegative and culture negative cats could have been exposed earlier in their lives to *Bartonella* species and cleared the infection, with either lack of antibody titers or titers below the positive threshold (score of 2 or more at 1:64 dilution). Additionally, the array was probed with 8 *Bartonella*-free cat sera (from cats never exposed to *Bartonella*) to establish non-specific reactivity. In total, microarray data from 132 cat sera were used to generate a profile of host reactivity to *B. henselae* infection. Representative images of protein microarrays probed with sera from *B. henselae*-infected and naïve cats samples are shown in Figure 1c and 1d. Signal intensities of duplicate spots were recorded and averaged for each antigen and for each cat serum, individually. Antigens were considered seroreactive if the average signal intensity exceeded the average signal intensity of the IVTT reaction without plasmid (No-DNA controls) plus 2.5-times the standard deviation. As expected, duplicate spots were highly correlative with a total R squared of 0.99, similar to our previous published results from a *Chlamydia trachomatis* protein microarray [32].

### Profile of humoral immune response to the *Bartonella henselae* proteome

**Figure 2:**
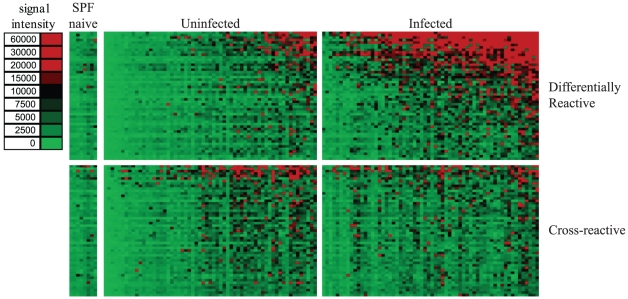
Probing a collection of *B. henselae* infected, uninfected, and SPF control cat sera. Arrays containing 1433 *B. henselae* proteins were probed with cat sera organized into 3 groups as described in the text. Heatmap showing normalized intensity with red strongest, bright green weakest, and black in between. Cat samples are in columns and sorted left to right by increasing average intensity to differentially reactive antigens, and antigens are listed in rows sorted by decreasing average seroreactivity of infected cats. Only seroreactive antigens are displayed (n = 105). Seroreactivity is higher in the infected cats against differentially reactive antigens, but equally reactive in the cross-reactive antigen set.

**Figure 3:**
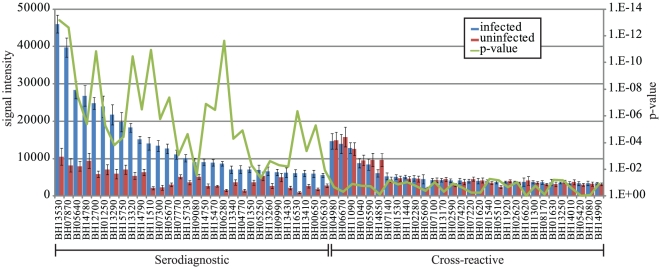
Discovery of cat serodiagnostic antigens. The 30 most reactive serodiagnostic and differentially reactive antigens are plotted on the x-axis. The mean seroreactivity of each antigen was compared between the infected (n = 62) and uninfected (n = 62) groups plotted in blue and red, respectively, with SEM. Corresponding p-values for each antigen are shown as a green line on the secondary y-axis.

The antibody response profile to *B. henselae* in its natural cat host is shown as a heatmap for both infected and uninfected cats (Fig. 2.). Of the total 1433 ORFs, 105 or 7.3% were found to be seroreactive. Cellular localization prediction of seroreactive ORFs revealed that 34 of 105 contained a signal peptide. Twenty-three were predicted to be localized to the cytoplasm, 21 to the cytoplasmic membrane, 9 to the outer membrane, 3 were periplasmic, and 49 were unable to be predicted by PSORTb. The mean reactivity for each protein on the array was compared between the infected and uninfected groups and plotted as a histogram (Fig. 3). Fifty-two antigens were differentially reactive (p<0.05), and 53 antigens were cross-reactive (p>0.05) between infected and uninfected cats. All of the sera reacted similarly to the cross-reactive antigens whether from infected or uninfected cats. Mean reactivity of seroreactive antigens was correlated to IFA score, and the Pearson's R was determined to be 0.70 for differentially reactive antigens and 0.17 for cross-reactive antigens, indicating a strong association with current IFA diagnostic assays and the identified differentially reactive antigens. A complete list of all seroreactive antigens is shown in Table S1. Twenty-four of the 62 seropositive cats were culture positive. The average seroreactivity from cats that were seropositive and culture positive was not significantly different from the average seroreactivity of cats that were seropositive and culture negative for either differentially reactive or cross-reactive antigens (Student's t-test p-value  = 0.75 and 0.36, respectively).

### *Bartonella henselae* type-specific immune response

**Figure 4:**
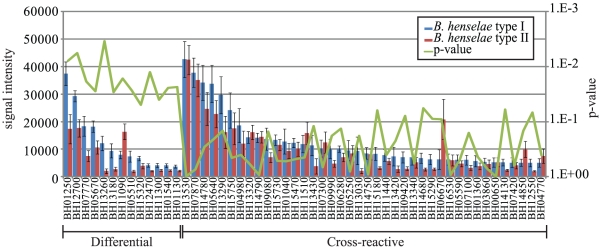
Differential seroreactivity of *B. henselae* type I and II cats. Mean seroreactivity of *B. henselae* type I (n = 10) and type II (n = 14) are plotted in blue and red, respectively. Corresponding SEM and p-values on the secondary y-axis are shown for each antigen. All nine differentially reactive antigens and the 30 most reactive cross-reactive antigens are plotted on the x-axis. Antigens are sorted left to right by decreasing seroreactivity of *B. henselae* type I infected cats.

Differential antibody profiles were investigated for type I and type II *B. henselae* (Fig. 4). Of the cat sera profiled by microarray, 10 were *B. henselae* type I and 14 were *B. henselae* type II. Thirteen antigens were statistically differentially reactive between these two groups for all 105 seroreactive antigens (Student's t-test p-value <0.05). The other 92 were equally reactive, including the two most reactive antigens (BH13530 (MopA) and BH07870 (LemA)). *B. henselae* type I cats were most significantly differentially reactive to BH01250 (CyoD) and BH13260 (VirB2, p = 5.5×10^−3^ and 5.0×10^−3^). BH01250 is the 6^th^ most reactive antigen, and BH13260 is the 23^rd^ most reactive antigen of the infected cat group. BH12700 (VceA) is the 5^th^ most reactive antigen of the infected cat group and had a mean signal intensity of 25k. The mean reactivity for *B. henselae* types I and II was 29 k and 21 k, respectively, suggesting that the distribution of type I and type II infected cats was evenly represented among the 62 infected cats. The same was true for the second most type-specific differentially reactive antigen (BH01250). BH01250 had a mean signal intensity of 24 k, and *B. henselae* types I and II had signal intensities of 37 k and 18 k, respectively. One antigen (BH11090) was found to be significantly more reactive in the type II infected cat group than in the type I infected group (p-value = 1.7×10^−2^).

### Validation of seroreactivity with immunostrips

**Figure 5:**
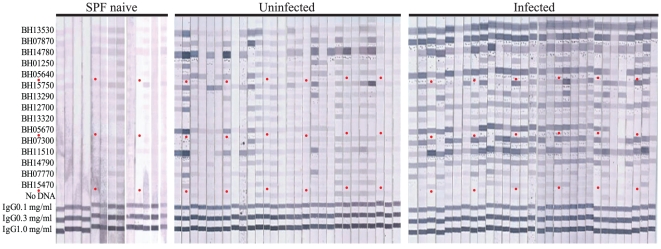
Immunostrips probing. Fifteen serodiagnostic antigens were printed onto nitrocellulose paper in adjacent stripes using a BioDot jet dispenser as described in Materials and Methods. Strips were probed with infected and uninfected sera diluted 1/200 followed by alkaline phosphatase conjugated secondary antibody and enzyme substrate. Weak reactivity in uninfected controls can be distinguished from the strong reactivity in the infected group. A red dot under the 5^th^, 10^th^, and 15^th^ antigen is indicated as a guide marker.

In order to validate the seroreactivity of the protein microarrays and to test the feasibility of using serodiagnostic antigens in an alternative analytical diagnostic assay, 15 antigens were printed onto nitrocellulose membranes, referred to as immunostrips. These 15 antigens were chosen for being highly significant and highly seroreactive by protein microarray. Individual immunostrips were probed with 30 infected and 28 uninfected cat sera chosen from the collection at random (Fig. 5). *Bartonella*-infected cat sera reacted strongly with the differentially reactive antigens, although the intensity pattern varied depending on the individual cat. Uninfected cat sera had low reactivity with these antigens, and produced a different pattern of reactivity than infected cats. Quantitative analysis of the immunostrips was used to directly compare seroreactivity of infected and uninfected cats on the immunostrips platform. The three most differentially reactive antigens in the protein microararay BH13530, BH07870, BH12700 (p-value = 7.0×10^−14^, 2.3×10^−13^, and 3.3×10^−8^, respectively) were also the most significantly different in the immunostrip, showing validation of the microarray and transference to a separate diagnostic platform (p-value  = 1.39×10^−14^, 1.73×10^−13^, and 4.0×10^−10^ respectively). Furthermore, the immunostrips were probed with SPF naïve sera to confirm and evaluate background reactivity in uninfected cats. As expected, SPF naïve cats displayed minimal reactivity to all bands, similar to most of the uninfected group.

### Serodiagnostic accuracy

**Figure 6:**
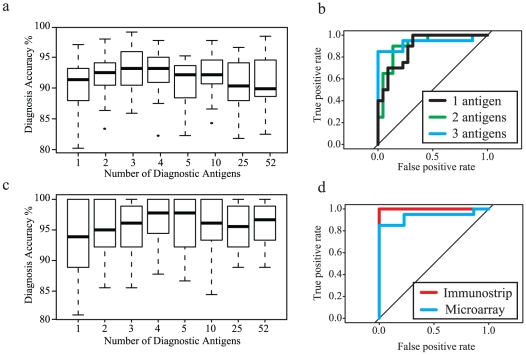
Multiple antigen classifier. The boxplots show classifiers with increasing number of serodiagnostic antigens. (a) Boxplots for the microarray classifier using the top 1, 2, 3, 4, 5, 10, 25, 52 antigens (b). The ROC curves were generated for each antigen set and a maximum predictive accuracy of 93%. ROC curves show that accuracy increases as multiple antigens are used to generate the classifier when increasing from 1 to 2 antigens, and from 2 to 3 antigens. (c) Boxplots for the classifier using the immunostrips are plotted. (d) A comparison of the ROC curves for the diagnostic accuracy of the microarray platform using 3 antigens compared to the ROC curve of immunostrips using 4 antigens.

In order to determine the diagnostic ability of the differentially reactive antigens, we used kernel methods and support vector machines [33], [34] to build linear and nonlinear classifier from microarray and immunostrips data. As such, we utilized the discriminatory power of multiple ORFs in order to assess their ability to separate uninfected from infected sera. Serodiagnostic antigens were ranked according to p-value with the top 3 antigens having p-values less than 7.0×10^−14^ (Table S1). We input the most significantly different antigens in sets of 1, 2, 3, 4, 5, 10, 25, and 52 antigens on the basis of p-value. The boxplots for these predictions were plotted and the results show that increasing the number of antigens from 1 to 2, and 2 to 3 produces an improvement in the classifier (Fig. 6). The classifier was able to accurately predict 93% of infected cats using 3 antigens. Increasing the diagnostic set from 3 to 4 antigens produced no increase in accuracy, and increasing past 4 antigens results in a reduction in accuracy due to over-fitting.

A subset of sera that was tested on the microarray platform was selected at random and demonstrated seroreactivity on the immunostrip platform. In order to determine the potential diagnostic utility of these differentially reactive antigens, quantified immunostrip results were used to build a classifier and accuracy was determined, as shown in Figure 6. As in the microarray classifier, increasing the number of antigens produced a more accurate diagnostic classifier until a peak was reached followed by a reduction in accuracy due to over-fitting. We found that the most accurate diagnostic ability utilized 4 antigens and produced a diagnostic accuracy capable of identifying 98% of the infected cats. Differences in accuracy and the number of antigens that increase the diagnostic accuracy between immunostrips and microarrays are an expected result from utilizing different platforms for diagnostics.

### Functional classification of the reactive antigens

We next classified the serodiagnostic and cross-reactive antigens according to their annotated and computationally predicted features. The summary of this analysis can be found in Table 1. The NCBI database of Clusters of Orthologous Groups of proteins (COGs) was used for annotation. Each COG consists of individual proteins or groups of paralogs from at least 3 lineages, and is comprised of 25 categories of functional definitions. Each protein in the database is assigned to one or more COGs, with a total of 1569 COGs assigned to the 1433 ORFs on the array. The results in Table 1 illustrate that reactivity is not evenly distributed between various COGs and some are much more likely to contain seroreactive ORFs. For example COG V, containing proteins involved in defense mechanisms, was found to be enriched for seroreactive ORFs (4.20 fold enrichment, p-value  = 2.9×10^−2^). In contrast, COG E (amino acid transport and metabolism) contained no seroreactive proteins, despite the presence of 100 COG E ORFs in our array. Underrepresentation was also found in proteins involved in transcription (1/78 or 1.3%, p-value = 3.9×10^−2^) and translation (2/138 or 1.4%, p-value = 2.9×10^−3^). Analysis of individual COGs showed that no category was entirely reactive. For instance, the most predictive COG (COG U - intracellular trafficking and secretion) contained only 23.5% seroreactive proteins (16/68, 3.30 fold enrichment, p-value = 1.0×10^−5^). The next most predictive COG (COG M - cell wall/membrane biogenesis) contained 17.7% seroreactive proteins (16/90, 2.49 fold enrichment, p-value = 3.8×10^−4^). Utilization of the COG database allowed characterization of functional group enrichment and under-representation; however, 292 *B. henselae* proteins were not defined by the COG database. Interestingly, this undefined category contained a significant number of seroreactive proteins (32/292, 1.54 fold enrichment, p-value = 7.7×10^−3^) compared to ORFs that were grouped into various COGs (80/1277, 0.88 fold enrichment, p-value = 7.7×10^−3^).

**Table 1:** COG enrichment table.

		Seroreactive				Differentially reactive				Cross-reactive			
NCBI COG definition	Total on array	hits		Fold Enrich	p-value	hits		Fold Enrich	p-value	hits		Fold Enrich	p-value
A - RNA processing and modification	0	0		0.00	1.0E+00	0		0.00	1.0E+00	0		0.00	1.0E+00
B - Chromatin structure and dynamics	0	0		0.00	1.0E+00	0		0.00	1.0E+00	0		0.00	1.0E+00
C - Energy production and conversion	71	8		1.57	1.6E−01	6	*	2.55	2.7E−02	2		0.74	1.0E+00
D - Cell cycle control, mitosis and meiosis	22	3		1.91	2.0E−01	1		1.37	5.3E−01	2		2.38	2.0E−01
E - Amino acid transport and metabolism	100	0	*	0.00	9.0E−04	0		0.00	7.4E−02	0	*	0.00	2.9E−02
F - Nucleotide transport and metabolism	45	1		0.31	3.7E−01	0		0.00	4.0E−01	1		0.58	1.0E+00
G - Carbohydrate transport and metabolism	48	2		0.58	5.8E−01	0		0.00	4.0E−01	2		1.09	7.1E−01
H - Coenzyme transport and metabolism	55	1		0.25	1.8E−01	0		0.00	2.6E−01	1		0.48	7.2E−01
I - Lipid transport and metabolism	38	1		0.37	5.2E−01	0		0.00	6.3E−01	1		0.69	1.0E+00
J - Translation	138	2	*	0.20	2.9E−03	1		0.22	8.1E−01	1		0.20	5.5E−02
K - Transcription	78	1	*	0.18	3.9E−02	1		0.39	5.1E−01	0		0.00	7.0E−02
L - Replication, recombination and repair	80	4		0.70	6.5E−01	0		0.00	1.1E−01	4		1.31	5.4E−01
M - Cell wall/membrane biogenesis	90	16	*	2.49	3.8E−04	9	*	3.02	2.1E−03	7		2.03	7.9E−02
N - Cell motility	4	0		0.00	1.0E+00	0		0.00	1.0E+00	0		0.00	1.0E+00
O - Posttranslational modification, protein turnover, chaperones	70	11	*	2.20	1.4E−02	7	*	3.02	7.0E−03	4		1.49	3.4E−01
P - Inorganic ion transport and metabolism	63	1		0.22	8.3E−02	0		0.00	2.7E−01	1		0.42	5.1E−01
Q - Secondary metabolites biosynthesis, transport and catabolism	12	0		0.00	1.0E+00	0		0.00	1.0E+00	0		0.00	1.0E+00
R - General function prediction only	152	5		0.46	6.6E−02	2		0.40	2.3E−01	3		0.54	3.6E−01
S - Function unknown	90	5		0.78	6.8E−01	2		0.67	7.7E−01	3		0.87	1.0E+00
T - Signal transduction mechanisms	39	0		0.00	1.1E+00	0		0.00	6.4E−01	0		0.00	3.9E−01
U - Intracellular trafficking and secretion	68	16	*	3.30	1.0E−05	8	*	3.55	1.3E−03	8	*	3.23	2.5E−03
V - Defense mechanisms	10	3	*	4.20	2.9E−02	2	*	6.03	4.1E−02	1		2.75	3.1E−01
W - Extracellular structures	4	0		0.00	1.0E+00	0		0.00	1.0E+00	0		0.00	1.0E+00
Y - Nuclear structure	0	0		0.00	1.0E+00	0		0.00	1.0E+00	0		0.00	1.0E+00
Z - Cytoskeleton	0	0		0.00	1.0E+00	0		0.00	1.0E+00	0		0.00	1.0E+00
Not in COGs	292	32	*	1.54	7.7E−03	16	*	1.65	2.9E−02	16		1.50	8.7E−02
**Total COGs**	**1569**	**112**				**55**				**57**			

Enrichment table of COG functional groups for seroreactive, serodiagnostic, and cross-reactive antigens. The numbers of annotated proteins printed on the array for each COG are totaled under “Total on array.” Seroreactive, serodiagnostic, and cross-reactive annotated proteins are totaled as “counts”. Asterisks denote significant values.

In addition to looking for COG enrichment, we also analyzed enrichment based on subcellular localization of proteins. Localization was predicted from ORF sequence with pSORTb software. From our entire ORF collection, pSORTb predicted 28 outer membrane proteins, of which 9 were seroreactive (32%). This was the most enriching feature based on localization (4.39 fold enrichment, p-value = 9.2×10^−5^). Conversely and as expected, we found pSORTb predicted cytoplasmic proteins were significantly underrepresented (0.63 fold enrichment, p-value = 2.9×10^−3^). Interestingly, the pSORTb predicted periplasmic proteins, as well as proteins in COG categories C, M, and O, were significantly enriched in only differentially reactive antigens, but not in cross-reactive antigens. These categories may prove useful for *in silico* prediction of potentially protective antigens and diagnostics. Proteins predicted to have isoelectric points between 5 and 7 are significantly underrepresented in the seroreactive group (0.69 fold enrichment, p-value = 1.5×10^−2^). This group is comprised of differentially reactive and cross-reactive proteins, in which only the cross-reactive proteins are significantly underrepresented (0.54 fold enrichment, p-value = 7.3×10^−3^), and would be poor targets for *in silico* predictors of diagnostic antigens. We found that more acidic proteins are less likely to be seroreactive and are more likely to be located in the cytoplasm, which may explain their underrepresentation. Proteins predicted to have isoelectric points between 5–7 were also significantly underrepresented in seroreactive and cross-reactive groups.

Some molecules can be expressed at high levels *in vivo* making them more likely targets for immune recognition, independent of their functional category. In order to determine the validity of this assumption, we looked at frequency distribution of codon adaptation index (CAI). Generally, higher CAI-values reflect potentially higher expression levels of an ORF. The CAI-values of *B. henselae* range from 0.27 to 0.71. While we found significance in ORFs that score in the middle on the CAI index range (from 0.4 to 0.5, fold enrichment 1.28, p-value =  3.4×10^−2^), we were surprised to find that antigens with high CAI values (which tend to be over-expressed) were not significantly enriched (Table 2). The bias for identifying highly expressed proteins discovered using 2D gels could be an inherent artifact not found using *in vitro* expression based platforms, like protein microarrays.

**Table 2:** Computationally predicted feature enrichment table.

		Seroreactive				Differentially reactive				Cross-reactive			
Computational Predictions	Total on array	hits		Fold Enrich	p-value	hits		Fold Enrich	p-value	hits		Fold Enrich	p-value
TMHMM = 0	1088	50	*	0.63	6.6E−05	27	*	0.68	1.8E−04	23	*	0.57	2.3E−07
TMHMM ≥1	345	55	*	2.18	6.6E−11	25	*	2.00	1.8E−04	30	*	2.35	2.5E−07
TMHMM ≥5	74	1	*	0.18	3.8E−02	0		0.00	1.7E−01	1		0.34	3.6E−01
TMHMM ≥10	0	0		0.00	1.0E+00	0		0.00	1.0E+00	0		0.00	1.0E+00
SignalP ≥0.7 (contains signal peptide)	194	34	*	2.39	3.0E−07	19	*	2.92	4.3E−06	15	*	1.94	8.6E−03
SignalP <0.7	1239	71	*	0.78	3.0E−07	33	*	0.73	1.7E−05	38	*	0.83	3.4E−03
PSORTb Cytoplasmic	502	23	*	0.63	2.9E−03	16		0.88	5.6E−01	7	*	0.38	3.8E−04
PSORTb CytoplasmicMembrane	229	21		1.25	2.7E−01	11		1.32	3.3E−01	10		1.18	5.7E−01
PSORTb Extracellular	2	0		0.00	1.0E+00	0		0.00	1.0E+00	0		0.00	1.0E+00
PSORTb OuterMembrane	28	9	*	4.39	9.2E−05	5	*	5.33	1.9E−03	4	*	3.59	2.3E−02
PSORTb Periplasmic	19	3		2.15	1.6E−01	3	*	4.71	2.3E−02	0		0.00	1.0E+00
PSORTb Unknown	653	49		1.02	8.4E−01	17		0.72	6.5E−02	32	*	1.32	3.4E−02
isoelectric point pI 0–5	82	9		1.49	1.9E−01	6		2.02	1.2E−01	3		0.99	1.0E+00
isoelectric point pI 5–7	511	26	*	0.69	1.5E−02	15		0.88	6.5E−01	11	*	0.54	7.3E−03
isoelectric point pI 7–9	323	26		1.10	5.5E−01	12		1.02	8.7E−01	14		1.17	5.0E−01
isoelectric point pI 9–14	517	44		1.16	2.1E−01	19		1.01	1.0E+00	25		1.31	1.1E−01
CAI 0.0–0.4	36	0		0.00	1.1E−01	0		0.00	6.3E−01	0		0.00	6.4E−01
CAI 0.4–0.5	512	48	*	1.28	3.4E−02	22		1.18	3.1E−01	26	*	1.37	4.2E−02
CAI 0.5–0.6	798	53		0.91	2.6E−01	27		0.93	5.7E−01	26		0.88	3.3E−01
CAI 0.7–1.0	87	4		0.63	4.0E−01	3		0.95	1.0E+00	1		0.31	3.7E−01
**Total ORFs**	**1433**	**105**				**52**				**53**			

Enrichment table of computationally predicted functional groups for seroreactive, serodiagnostic, and cross-reactive antigens. The numbers of proteins printed on the array for each predicted feature are totaled under “Total on array.” Seroreactive, serodiagnostic, and cross-reactive proteins with predicted features are totaled as “counts”. Asterisks denote significant values.

## Discussion

Our results represent a large-scale analysis of *B. henselae* proteins that are immunogenic in the context of naturally acquired feline infection. We have constructed the first protein microarray for *B*. *henselae*, which allowed us to assess the humoral immune response to *B. henselae* from 62 naturally infected cats. The seroreactive antibody profile revealed many unknown seroreactive antigens, and confirmed previously identified ones. In this research, we sought to identify potentially protective and diagnostically relevant *B. henselae* antigens, and to understand the repertoire of the humoral immune response to infection in the natural feline host reservoir. In a genetically diverse population, the antibody repertoire is expected to vary among individual cats. Despite this diversity, the subset of differentially reactive antigens we identified provides a predictive accuracy rate of 93% for diagnosis of *B. henselae* exposed cats using the microarray (and 98% with the immunostrips), and it is likely that this set of antigens will form the basis of new and more accurate serodiagnostic assays for *Bartonella* exposure. Additionally, utilization of these antigens in alternative platforms (e.g., ELISA format) may provide an easy, universal, and rapid diagnostic assay. Importantly, some of the antigens we have discovered could be ideal candidates for generating subunit vaccines that protect cats from infection, thus limiting the morbidity and mortality of incidental human infections. All differentially reactive antigens, as well as the level of differential seroreactivity are not expected to correlate with protection, and empirical evaluation of selected, differentially reactive antigens will have to be determined. Of note, vaccination with facultatively intracellular pathogens, like *B. henselae,* could also depend on effective T-cell memory for mediating host defense and production of specific antibodies. Our study investigated the immune response of cats at a single time point, and we were not able to document previous infection of these cats by other *Bartonella* species or genotypes, including *B. clarridgeiae* or *B. koehlerae*, for which some cross-reactivity may occur [1]. Therefore, in some cats, the antibody profile could represent undetected coinfection or superinfection. In fact, protection of cats from infection with both *B. henselae* type I and type II would be ideal, because both types cause human zoonotic infections.

The most differentially reactive antigens on the immunostrips were able to accurately predict 98% of infected cats using 3 antigens (MopA, VceA, and LemA). Inclusion of the next highest ranked antigen, VirB8 (a type IV secretion system [T4SS] molecule), did not significantly improve the predictive accuracy. Antigens that are involved in T4SS are key factors in mediating *Bartonella*-host cell interactions, and five T4SS components are found in the serodiagnostic set (TrwE, TrwG, VirB2, VirB8, and VirB10). Interestingly, no T4SS molecules were found in the cross-reactive antigen set. T4SS is comprised of a protein complex that is used to transport effector molecules directly into the host target cell. T4SS molecules are attractive targets for the development of new therapeutic agents, and investigation of monoclonal antibody therapy or small molecule inhibitors to block the secretion of virulence factors could provide protection or limit disease. The cross-reactive protein ParA is a plasmid partitioning protein, and the gene encoding this protein was previously identified as the only unique gene absent in *B. quintana*, although it is present in the genomes of both *B. koehlerae* and *B. henselae* [35]. Lindroos, *et al.*, believe it is a pseudogene and not involved in host specificity, because the gene is shorter than normal in *B. henselae* and it is not flanked by a homolog to *parB,* as are most other *parA* genes. The role of this protein in association with the feline pathogen remains to be investigated, as well as its role in establishing acute and chronic infection. BH12700 (VceA, a multidrug resistance gene), and BH13260 (VirB2, a T4SS molecule), are both differentially reactive between genotypes I and II, and could facilitate an understanding of the differences between the two types. Experimental infection with *B. henselae* type I or type II in SPF cats provides complete protection against a challenge with the same genotype [3], [36]. However *B. henselae* type I provides partial protection from a challenge with *B. henselae* type II. On the contrary cats infected with *B. henselae* type II were not protected when challenged with the heterologous strain, *B. henselae* type I [37]. The two most differentially reactive antigens are equally reactive in both types I and II, and subunit vaccines including these antigens should be ideal candidates for protection against both types I and II of *B. henselae*. Further investigation of the antibody profiles of protected and non-protected SPF cats could provide insight into individual antigens or profiles of antigens responsible for mediating cross-protection against both types, as our study was a cross-sectional study and some of the bacteremic cats may have had a previous infection with the other *B. henselae* genotype or even with other *Bartonella* species leading to production of cross-reactive antibodies [37].

We found that reactivity was not evenly distributed across the proteome and no individual category was completely seroreactive (Table 1). Cross-reactive and differentially reactive antigens are selectively enriched or underrepresented from specific functional categories. As expected, outer membrane proteins are more likely to be seroreactive (4.39 fold enrichment, p-value = 9.2×10^−5^) and account for 8.6% of reactive antigens, but represent only 2.0% of the proteins on the array. As expected, antigens predicted to be cytoplasmic are significantly underrepresented (comprising only 4.6% of total seroreactive antigens, but constitute 34.8% of the proteins on the array). While these results classifying antigenicity by protein localization are consistent with expectations, they provide a quantitative and more informative understanding than previously reported. Importantly, there is no category that is entirely reactive, although there are some categories that are entirely unreactive. For example, there are 100 ORFs that are involved in amino acid transport and metabolism, but none of these antigens were seroreactive. Moreover, we found ORFs involved in translation and transcription to be significantly less likely to be seroreactive (p-value = 2.9×10^−3^ and 3.9×10^−2^, respectively). Antigens predicted to contain at least one transmembrane domain were highly significantly enriched on the array (2.18 fold enrichment, p-value = 6.6×10^−11^). Conversely, antigens that were predicted to not contain a transmembrane domain were significantly underrepresented (0.063 fold enrichment, p-value 6.6×10^−5^). Identification of categories that are non-reactive is important for understanding immune evasion and pathogenicity, and is also valuable for developing exclusion criteria of *in silico* prediction algorithms. Similarly, cross-reactive antibodies may target identical proteins (or common epitopes) of both related and unrelated bacteria, and are thought to exist as a consequence of an indiscriminate or broad antibody response against numerous bacteria, whether symbiotic, environmental, or other. Moreover, cross-reactivity does not need to occur between closely related organisms, but can also occur between phylogenetically distant species. In this report, we identify numerous cross-reactive antigens in a cat population, which may be important for improved diagnostics and vaccine development. These results are consistent with our previously published results detailing the seroreactive profile of *Burkholderia pseudomallei* [21], another proteobacteria member. We again found significant enrichment of surface, chaperones, and PSORTb-predicted extracellular and outer membrane proteins. Moreover, inclusion of a predicted signal peptide or transmembrane domain were both enrichment features. Proteins predicted by PSORTb to be cytoplasmic or of unknown localization, and proteins predicted to not contain transmembrane domains were also significantly underrepresented in our previous profiling of seroreactive antigens from *Burkholderia* infected patients [21].

A comprehensive profile of the antibody repertoire to an infectious agent can provide new insight into disease pathogenesis. The data reported here provide specific insight into the antigens that are recognized by the humoral immune response after natural infection(s) in a naturally infected cat population. We found that the humoral immune response is not stochastic, and targets a diverse collection of antigens. The results presented here confirm that proteomic features can help predict seroreactive antigens, but these predictions are imperfect because the majority of proteins predicted are nonreactive. The empirically determined antibody repertoire against the comprehensive *B. henselae* proteome highlights the need for an improved understanding of seroreactivity determinants. Finally, our characterization of the feline antibody repertoire generated during *B. henselae* infection provides novel insight and utility in diagnostics, vaccine development, and in understanding the host-pathogen interaction of a zoonotic infectious agent.

## Supporting Information

Table S1 Seroreactive antigen list.
(0.05 MB XLS)
